# Genomic characterization of Alphacoronavirus from *Mops condylurus* bats in Nigeria

**DOI:** 10.1016/j.virusres.2023.199174

**Published:** 2023-07-24

**Authors:** Uwem George, Oluwadamilola George, Judith Oguzie, Oluwadamilola Osasona, Babatunde Motayo, Joshua Kamani, Philomena Eromon, Onikepe Folarin, Anise Happi, Isaac Komolafe, Christian Happi

**Affiliations:** a African Centre of Excellence for Genomics of Infectious Diseases, Redeemer's University, Ede, Osun State, Nigeria; b Department of Biological Sciences, Faculty of Natural Sciences, Redeemer's University, Ede, Osun State, Nigeria; c National Veterinary Research Institute, Vom, Plateau State, Nigeria; d Department of Medical Microbiology, Federal Medical Centre, Abeokuta, Nigeria; e Parasitology Division National Veterinary Research Institute NVRI PMB 01, Vom, Plateau state Nigeria

**Keywords:** Alphacoronavirus, Nigeria, Molossidae, Metagenomics, Recombination

## Abstract

Coronaviruses (CoVs) are responsible for sporadic, epidemic and pandemic respiratory diseases worldwide. Bats have been identified as the reservoir for CoVs. To increase the number of complete coronavirus genomes in Africa and to comprehend the molecular epidemiology of bat Alphacoronaviruses (AlphaCoVs), we used deep metagenomics shotgun sequencing to obtain three (3) near-complete genomes of AlphaCoVs from *Mops condylurus* (Angolan free-tailed) bat in Nigeria. Phylogenetic and pairwise identity analysis of open reading frame 1ab (ORF1ab), spike (S), envelope (E), membrane (M) and nucleocapsid (N) genes of AlphaCoV in this study to previously described AlphaCoVs subgenera showed that the Nigerian AlphaCoVs may be members of potentially unique AlphaCoV subgenera circulating exclusively in bats in the *Molossidae* bat family. Recombination events were detected, suggesting the evolution of AlphaCoVs within the *Molossidae* family. The pairwise identity of the S gene in this study and previously published S gene sequences of other AlphaCoVs indicate that the Nigerian strains may have a genetically unique spike protein that is distantly related to other AlphaCoVs. Variations involving non-polar to polar amino acid substitution in both the Heptad Repeat (HR) regions 1 and 2 were observed. Further monitoring of bats to understand the host receptor use requirements of CoVs and interspecies CoV transmission in Africa is necessary to identify and prevent the potential danger that bat CoVs pose to public health.

## Highlights

• First report of a near-complete genome of bat Alphacoronavirus in Nigeria.
• Unique AlphaCoV subgenera circulating in the *Molossidae* bat family.
• Evidence of Alphacoronaviruses recombination within the *Molossidae* bat family.
• Nigerian AlphaCoV strains have a genetically distinct spike protein.
• Binding sites distinct from the motifs used for ACE2 and hAPN binding.

## 1 Introduction

In recent years, global epidemiological monitoring for coronavirus diversity among bats has expanded rapidly as a result of emerging coronavirus disease outbreaks such as SARS-CoV in China in 2002 (Ksiazek et al., 2003), MERS-CoV in the Arabian Peninsula in 2012 (Zaki et al., 2012) and SARS-CoV-2 in late 2019 (Zhu et al., 2020).

Coronaviruses (CoVs) are one of the largest RNA viruses with genome sizes ranging between 26–32 kilobases. The genome consists of a single-stranded positive-sense RNA cleaved to form the structural and non-structural proteins. The structural proteins described in coronaviruses include the spike (S), envelope (E), membrane (M) and nucleocapsid (N) genes respectively (Lai & Cavanagh, 1997; Masters, 2006). The S protein, among them, is crucial for the viral invasion and cell infection processes. Furthermore, coronaviruses have been shown to exhibit a high rate of recombination due to their distinct viral replication mechanism (Lai & Cavanagh, 1997; Masters, 2006; Pollett et al., 2021; Su et al., 2016), which in combination with the infidelity of their RNA polymerase, may enable their evolution and spread to new hosts and ecological regions. (Cui et al., 2019; Ruiz-Aravena et al., 2022; Xiao et al., 2020). On the other hand, the nsp14 of CoVs has been shown to encode a unique proofreading system during RNA synthesis, thereby reducing the rate of mutations due to RdRp errors during replication (Minskaia et al., 2006).

CoVs belong to the family *Coronaviridae* and have been divided into four genera: *Alphacoronavirus* (AlphaCoV), *Betacoronavirus* (BetaCoV), *Gammacoronavirus* and *Deltacoronavirus* (Weiss & Navas-Martin, 2005; Woo et al., 2009) of which only AlphaCoV and BetaCoV have been documented in bats (Woo et al., 2009). The International Committee on Taxonomy of Viruses (ICTV) (https:// talk.ictvonline.org/taxonomy/) species demarcation criteria for CoVs are based on members of the same species sharing more than 90% of their amino acid sequence identity in the ORF1ab polyprotein domains. Based on these criteria, the AlphaCoV genus is further divided into 15 subgenera with species members infecting a wide range of mammals including humans, bats, dogs, cats and pigs (De Sabato et al., 2019; Hossain et al., 2021; Muzeniek et al., 2022; Santana-Clavijo et al., 2020; van der Hoek et al., 2004; Wang et al., 2019). Human alphacoronaviruses (HCoV-NL63 and HCoV-229E) with the ability to spread from person to person have been associated with respiratory infections, sometimes resulting in severe respiratory pathologies in immunocompromised individuals, the elderly and children (Chiu et al., 2005; van der Hoek et al., 2006, 2004).

Recently, novel AlphaCoV strains have been detected in African bat species in the *Molossidae* family in Kenya, Eswatini and the Democratic Republic of Congo (Kuchinski et al., 2022; Shapiro et al., 2021; Tao et al., 2012). Interestingly, coronavirus-positive bats were captured around roosts near houses, churches, or human settlements (Shapiro et al., 2021) and to date, these strains have not been assigned to any subgenera. There are still unknown numbers of bat species in Africa that harbour CoVs with numbers rising in direct proportion to the level of surveillance. In this study, we present the identification and genomic characterization of additional bat AlphaCoVs from *Mops condylurus* bats in Nigeria that provide further insight into the diversity and intricate evolutionary history of AlphaCoVs within bat species in the family *Molossidae*. This is critical for understanding the cross-species transmission and evolutionary trends of AlphaCoVs.

## 2 Materials and methods

### 2.1 Sampling and RNA extraction

Bat samples (pooled oral/rectal swab) analysed in this study were taken from *Mops condylurus* (Angolan free-tailed bat) and *Chaerephon spp.* (Little free-tailed bats) trapped in 2020 and 2021 from the ceiling of a residential building in Gboko, Benue state, and also from a primary healthcare (PHC) facility in Paiko, Niger state, Nigeria (supplementary Fig. 1). The samples all tested positive for unclassified AlphaCoV genera using a nested RT-PCR pan-coronavirus assay (George et al., 2022). Ten RT-PCR positive samples with sufficient volume (Table 1) were chosen for in-depth genome sequencing and NGS analysis.

**Table 1:** Summary of alphacoronavirus reads and additional viruses detected in the bat samples.

Sample ID	Sex	Age	Total reads	AlphaCoV reads	AlphaCoV reads %	Nucleotide length	Number of Contigs	Other viruses in the sample
Bat_GB04NGR^a^	Male	Juvenile	170,878	0	0.0	NA	0	1
Bat_GB09NGR^a^	Female	Adult	381,938	42,243	11.06	27,932	14	2, 3, 4, 5, 6, 7, 8
Bat_GB010NGR^a^	Female	Adult	980,132	0	0.0	NA	0	6, 9, 10, 11, 12
Bat_GB012NGR^a^	Female	Juvenile	516,000	35,906	6.96	27,544	3	13
Bat_GB013NGR^a^	Female	Adult	2269,408	19,170	0.84	27,968	1	7, 9, 10, 11, 13, 14, 15, 16, 17, 18, 19, 20, 21
Bat_NG017NGR^b^	Male	Adult	150,242	0	0.0	NA	0	NA
Bat_NG019NGR^b^	Female	Adult	297,842	0	0.0	NA	0	NA
Bat_NG022NGR^b^	Female	Adult	151,812	0	0.0	NA	0	NA
Bat_NG024NGR^b^	Female	Adult	229,088	0	0.0	NA	0	NA
Bat_NG033NGR^b^	Female	Adult	388,590	0	0.0	NA	0	NA

Abbreviations: Alphacoronavirus, AlphaCoV; NA, Not Available.

^b^Sample from *Chaerephon sp*.

^a^Sample from *Mops condylurus* bat.

Other viruses are present in the sample with numbers from 1 to 21 as follows: 1 = Bat picornavirus, 2 = Bastrovirus, 3 = Parechovirus, 4 = Sapovirus, 5 = Guadeloupe mosquito virus, 6 = Southern cowpea mosaic virus, 7 = Hubei permutotetra-like virus, 8 = Drosophila A virus, 9 = Arivirus, 10 = Calhevirus, 11 = Solenopsis invicta virus, 12 = Unclassified picornavirus, 13 = Culex Daeseongdong-like virus, 14 = Riboviria sp, 15 = Bunderbeg virus, 16 = Euprosterna elaeasa virus, 17 = Hangzhou tombus-like virus, 18 = Hubei picorna-like virus, 19 = Monexpicovirus, 20 = Shuango permutotetra-like virus 1, 21 = Spissistilus festinus virus 1.

Viral RNA was extracted from the samples using a QIAamp® Viral RNA extraction kit (Qiagen®, Hilden, Germany) with an elution volume of 60 μL according to the manufacturer's instructions and stored at −80°C until further processed.

### 2.2 Library preparation and sequencing

Library preparation was performed using an unbiased next-generation RNA sequencing method that allows for intra-host variation calls and de novo assembly of viral genomes collected from clinical and biological sources as previously described (Matranga et al., 2016; Oguzie et al., 2022). Briefly, after elution, the RNA was subjected to turbo DNase treatment to eliminate any contaminating DNA. Thereafter, cDNA was carried out using a Superscript III Synthesis kit (Invitrogen) with random primers. The Illumina Nextera XT kit was used to make sequencing libraries. After that, paired-end sequencing was performed on an Illumina Miseq platform with the Illumina MiSeq Reagent Kit v2 (500 cycles).

### 2.3 Read processing and phylogenetic analysis

Raw reads were checked for quality and trimmed to remove sequencing adapters using trimmomatic (Bolger et al., 2014), after which the Premonition metagenomics pipeline from Microsoft (https://innovation.microsoft.com/en-us/premonition) was used initially to process the trimmed raw reads. MetaSPAdes was used to do a de novo assembly of samples with alphacoronavirus reads into contigs (Nurk et al., 2017). Alphacoronavirus contigs were searched for using the BLASTn tool (Chen et al., 2015) on the NCBI database. Assembled alphacoronavirus genomes were annotated using Core Sequence IDentifier (CORSID) (https://github.com/elkebir-group/CORSID) (Zhang et al., 2022). A dataset of complete or near-complete genome reference nucleotide sequences representing all the classified sub-genera in the *Alphacoronavirus* genus was obtained from the NCBI Virus database together with the top five (5) sequences from the BLASTn search that showed the highest nucleotide similarity to the strains analysed in this study (supplementary Table 1). Amino acid sequences from selected AlphaCoV sub-genera and sequences from this study were also aligned to determine the pairwise identity using the Sequence Demarcation tool (SDT) (Muhire et al., 2014).

Multiple alignments of nucleotide sequences and deduced amino acids were performed using MAFFT online service (https://mafft.cbrc.jp/alignment/server/) (Katoh et al., 2019). The maximum likelihood (ML) tree was constructed using IQ-TREE v1.6.12 (Nguyen et al., 2015) with ModelFinder (Kalyaanamoorthy et al., 2017) and ultrafast bootstrap (1000 replicates) (Hoang et al., 2018). The tree was visualised using Interactive Tree of Life (iTOL) v5 (Letunic & Bork, 2021).

### 2.4 Discrete phylogeographic analysis of alphacoronaviruses

Using the entire ORF1ab coding sequence, phylogenetic trees were constructed using Bayesian inference by Markov chain Monte Carlo (MCMC), which was implemented in BEAST version 2.5 (Bouckaert et al., 2019). We partitioned the coding genes into first+second and third codon sites and thereafter, applied the Hasegawa-Kishino-Yano (HKY+G) substitution model with gamma-distributed rate heterogeneity among sites to each partition (Hasegawa et al., 1985).

For the final analysis, a relaxed clock with a Gaussian Markov Random Field Skyride plot (GMRF) coalescent prior was selected. A 10% burn-in was applied to the MCMC chain, which had a setting of 100 000 000 states. The mean time of the most recent common ancestor (TMRCA) and the highest posterior density regions at 95% (HPD) was estimated. Results were visually displayed using Tracer v1.8 (http://tree.bio.ed.ac.uk/software/tracer/), and all effective sampling size ESS values were >200, indicating adequate sampling. Using Tracer v. 1.8, a Bayesian Skyride analysis was performed to visualize the evolutionary epidemic history. We utilized the discrete-trait model in BEAST version 2.5 for the reconstruction of the ancestral-state phylogeographic transmission across AlphaCoV subgenera (Bouckaert et al., 2019). The most significant historical dispersal routes for the spread of AlphaCoV across different subgenera were also investigated using the Bayesian stochastic search variable selection (BSSVS) approach (Lemey et al., 2009). The tree was visualized using the ggtree R package (Yu et al., 2017).

### 2.5 Recombination analysis

Nine alternative detection methods, including RDP, GENECONV, BootScan, MaxChi, Chimaera, 3Seq, PhylPro, LARD and SiScan, were used with default settings to test for the presence of recombination using the recombination detection program 4 (RDP4) (Martin et al., 2017). Only recombination occurrences predicted by at least six detection methods were considered reliable.

### 2.6 Sequence analysis of unclassified AlphaCoV spike proteins

The ProtParam and ProtScale tools on the ExPASy Server (accessible at https://web.expasy.org/protparam/) were used to calculate the physical and general biological properties of the unclassified AlphaCoV S proteins. Antigenic epitopes were predicted using a previously described approach (Kolaskar & Tongaonkar, 1990) with an antigen prediction tool (accessible at http://imed.med.ucm.es/Tools/antigenic.pl). We aligned the unclassified AlphaCoV spike receptor binding domain, S1 C-terminal domain and Heptad Repeat regions 1 and 2 with two reference human alphacoronaviruses: HCoV-NL63 (which uses angiotensin-converting enzyme 2 [ACE2]) and HCoV-229E (which uses human Aminopeptidase N [hAPN]) to determine the presence of amino acid substitutions and conserved regions that may predict possible binding to human receptors.

## 3 Results

### 3.1 NGS data analysis and Genomic organisation

Only three out of the ten AlphaCoV RT-PCR positive bat samples chosen for in-depth genome sequencing had AlphaCoV reads ranging from 0.84% to 11.06%, of which three near-complete genomes were successfully assembled (Table 1). We also assembled 15 partial genomes (13 contigs from sample GB09 with sizes ranging between 1000–21,000 nt and two contigs from sample GB012 with sizes ranging between 800–1000 nt). The three near-complete genomes possessed genome sizes of 27,932 for GB09-NGR_2020, 27,544 for GB012-NGR_2020 and 27,968 for GB013-NGR_2020, with GC content of 43.58%, 43.26% and 43.52 respectively (Table 1).

The BLASTn search of the assembled Nigerian strains with those available online showed similarity (95.01%-95.85%) with unclassified AlphaCoV strains detected in the *Molossidae* bat family from Kenya in Eastern Africa (KY22/2006-HQ728486) and Eswatini in Southern Africa (Bat151/Eswatini/2014-OL807610 and Bat143/Eswatini/2014-OL807601). Similar to other AlphaCoV species, their genomes were organized into eight ORFs and two non-translated termini in the following order: 5′ terminus-ORF1a-ORF1b-Spike-ORF3-Envelope (E)-Membrane (M)-Nucleocapsid (N)-ORFx-3′ terminus (Table 2). Sequence prediction of putative transcription regulating sequence (TRS) showed that the alphacoronaviruses detected in this study had the core sequence of the TRS (CTAAAC) only in the M, N and ORFX genes while CTAAAT/CTAAAC, CGTTAC and CTCTAC were observed in the S, ORF3 and E genes respectively (Table 2).

**Table 2:** Location of predicted open reading frames and transcription regulating sequences.

AlphaCoV Isolate	ORF	ORF position		ORF length (nt)	Putative TRS		
		ORF start	ORF end		Core start	Core end	TRS
GB09-NGR_2020	ORF1a	297	12,555	12,258			
	ORF1b	12,555	20,549	7994			
	S	20,553	24,624	4071	20,540	20,560	TCAACTAAATAAAATGTTTC
	ORF3	24,626	25,295	669	24,591	24,603	-CAACGTTACGAA——-
	E	25,281	25,503	222	25,268	25,280	-CAACTCTACGAA——-
	M	25,517	26,201	684	25,501	25,511	—TCTAAACGAA——-
	N	26,214	27,474	1260	26,203	26,211	–AACTAAAC———-
	ORFx	27,492	27,720	228	27,478	27,488	TCAACTAAAC———-
GB12-NGR_2020	ORF1a	302	12,092	11,790			
	ORF1b	12,092	20,086	7994			
	S	20,090	24,161	4071	20,077	20,097	TCAACTAAATAAAATGTTTC
	ORF3	24,163	24,832	669	24,128	24,140	-CAACGTTACGAA——-
	E	24,818	25,040	222	24,805	24,817	-CAACTCTACGAA——-
	M	25,054	25,738	684	25,038	25,048	—TCTAAACGAA——-
	N	25,751	27,011	1260	25,740	25,748	–AACTAAAC———-
	ORFx	27,029	27,257	228	27,015	27,025	TCAACTAAAC———-

GB13-NGR_2020	ORF1a	309	12,567	12,258			
	ORF1b	12,567	20,561	7994			
	S	20,565	24,633	4068	20,552	20,561	TCAACTAAA—-
	ORF3	24,635	25,304	669	24,600	24,612	-CAACGTTACGAA
	E	25,290	25,512	222	25,277	25,289	-CAACTCTACGAA
	M	25,526	26,210	684	25,509	25,520	–GTCTAAACGAA
	N	26,223	27,483	1260	26,212	26,220	–AACTAAAC—
	ORFx	27,501	27,729	228	27,487	27,496	TCAACTAAA—-

N/B: TRS = Transcription regulating sequences and similar nucleotides to the leader TRS are indicated by dashes (-); nt = Nucleotide.

Pairwise identity results of ORF1ab, S, E, M and N genes using the Sequence Demarcation tool showed a high difference between the Nigerian strains (GB09-NGR_2020 and GB013-NGR_2020 genomes) and previously characterized reference AlphaCoV subgenera. In contrast, only unclassified AlphaCoVs recently detected in the bat family *Molossidae* (Bat143/Eswatini/2014 [OL807609], Bat151/Eswatini/2014 [OL807610]-*Chaerephon pumilus* bat and CDAB0492R [ON313747.1]-*Mops condylurus* bat) possessed high sequence similarity (80%-96%) in the ORF1ab, E, M and N genes. The most striking difference between GB09-NGR_2020 and GB013-NGR_2020 genomes was observed in their S proteins, which shared <77% amino acid identity with the S proteins of other alphacoronaviruses (Table 3).

**Table 3:** Comparison of genome sizes and amino acid identities between predicted proteins of GB09-NGR-2020, GB013-NGR-2020 and other AlphaCoV based on Sequence Demarcation Tool (SDT).

AlphaCoV		Genome size (Base pair)	Pairwise amino acid identity (%)									
			GB09-NGR-2020					GB013-NGR-2020				
Subgenera	Reference sequence (Accession number)		ORF1ab	S	E	M	N	ORF1ab	S	E	M	N
**Decacovirus**	HKU10_CHN/2005 (NC_018871.1)	28,494	71.37	64.13	60.91	74.55	63.33	71.39	63.43	60.91	75.29	63.14
**Duvinacovirus**	HCoV-229E/2000 (NC_002645.1)	27,317	68.22	61.73	63.58	64.36	61.85	68.28	60.68	63.58	64.36	60.92
**Setracovirus**	HCoV-NL63/2003 (NC_005831.2)	27,553	68.68	61.86	65.96	64.08	60.28	68.58	62.10	65.96	63.93	60.30
**Myotacovirus**	BtMr-SAX2011 (NC_028811.1)	27,935	68.63	62.34	54.54	63.07	60.62	68.58	62.63	54.54	63.52	60.09
**Minunacovirus**	HKU8_HK (NC_010438.1)	28,773	69.83	65.76	71.57	74.55	66.54	69.68	65.57	71.57	74.85	66.41
**Nyctacovirus**	BtNv-SC2013 (NC_028833.1)	27,783	69.31	62.86	56.56	73.69	62.60	69.38	63.22	56.56	73.84	63.68
**Minacovirus**	FRCoV-NL/2010 (NC_030292.1)	28,434	61.62	61.93	50.26	59.75	58.99	61.73	61.57	50.26	59.90	58.77
**Colacovirus**	CDPHE15/USA/2006 (NC_022103.1)	28,035	69.14	62.51	61.61	65.77	63.48	69.01	61.80	61.61	65.62	63.48
**Pedacovirus**	PEDV/2001 (NC_003436.1)	28,033	69.38	61.68	56.56	73.51	63.22	69.20	61.12	60.10	73.80	63.75
**Tegacovirus**	PUR46-MAD/2000 (NC_038861.1)	28,586	64.79	61.56	53.80	58.28	58.67	64.60	61.23	53.80	58.28	40.98
**Rhinacovirus**	HKU2_CHN (NC_009988.1)	27,165	67.83	60.64	59.48	65.47	70.19	67.68	60.71	59.48	65.77	70.19
**Luchacovirus**	Lucheng-19/2013 (NC_032730.1)	28,763	64.10	59.82	54.82	60.39	55.57	63.93	59.82	54.82	60.39	56.12
**Soracovirus**	Shrew-CoV/Tibet2014 (KY370053)	27,102	61.22	59.94	53.26	55.67	58.56	61.43	60.31	53.26	55.52	58.55
**Sunacovirus**	Xingguo-74/2015 (NC_048211.1)	25,984	62.23	60.23	54.16	58.78	56.98	64.60	59.38	54.16	58.93	56.66
**Unclassified AlphaCoV (This study)**	GB012-NGR-2020	27,544	97.85	97.63	99.49	97.17	98.44	97.92	96.33	99.49	96.87	97.83
**Unclassified AlphaCoV**	Bat143/Eswatini/2014 (OL807609)	27,956	95.86	73.11	80.80	90.02	81.14	95.82	73.47	80.80	90.02	81.29
**Unclassified AlphaCoV**	Bat151/Eswatini/2014 (OL807610)	28,061	95.85	73.19	81.31	90.02	81.05	95.87	73.54	81.31	90.02	81.21
**Unclassified AlphaCoV**	CDAB0492R (ON313747.1)	27,989	96.08	77.07	96.96	98.36	96.88	96.12	76.72	96.96	98.21	96.96

### 3.2 Phylogenetic and discrete phylogeographic analysis

**Fig. 1:**
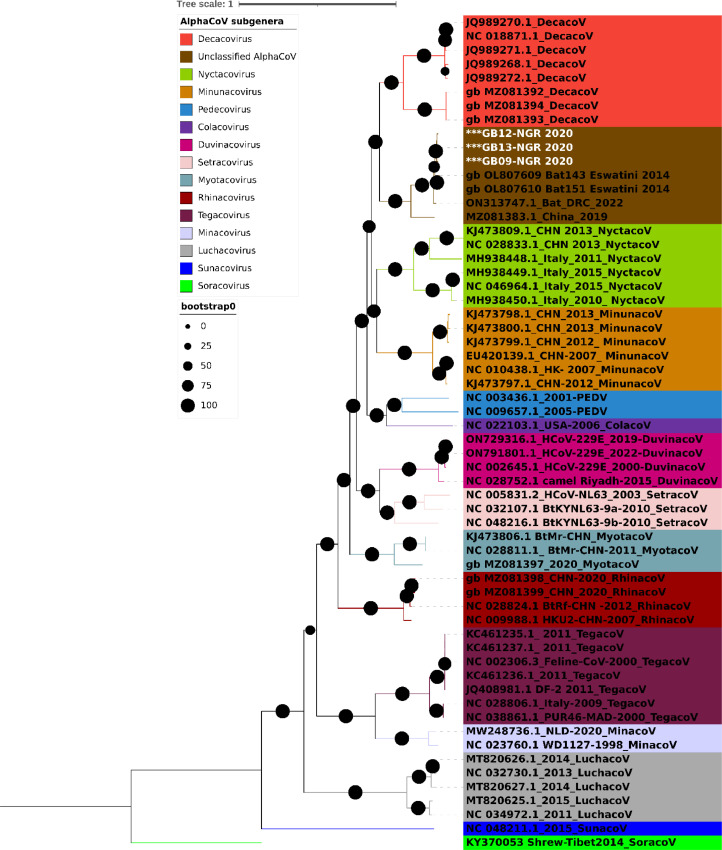
Maximum likelihood tree with ModelFinder based on complete ORF1ab sequences, with 1000 bootstrap replications. All AlphaCoV subgenera are colour-coded, as shown in the legend. AlphaCoV strains reported in this study are asterisked and highlighted in white. The Interactive Tree of Life (iTOL) v5 with midpoint rooting was used to visualise the tree. The Best-fit model according to BIC was GTR+F+I+G4.

A phylogenetic tree of the entire ORF1ab gene was constructed to examine the relationship between the sequences from this study and the AlphaCoV strains that have been previously discovered worldwide. The ML tree showed that strains detected in *Mops condylurus* bats in this study clustered with unverified Alphacoronavirus (CDAB0492R [ON313747.1]) detected in *Mops condylurus* bats in Democratic Republic of Congo (DRC) in Central Africa in 2022, *Chaerephon pumilus* bat alphacoronavirus (Bat143/Eswatini/2014 [OL807609] and Bat151/Eswatini/2014 [OL807610) detected in *Chaerephon pumilus* bats from Eswatini in Southern Africa in 2021 and *Alphacoronavirus sp.* strain bat/Yunnan/CpYN11/2019 (MZ081383) reported in China in 2021, forming a monophyletic clade (Fig. 1). The clustering of the Nigerian strains with the DRC, Eswatini and China strains was confirmed by the ML trees constructed using the predicted protein sequences of S, E, M, and N. (Supplementary Fig. 2). The consistent monophyletic clustering of the Nigerian AlphaCoV sequences suggest the sampled lineage was established by a single introduction

**Fig. 2:**
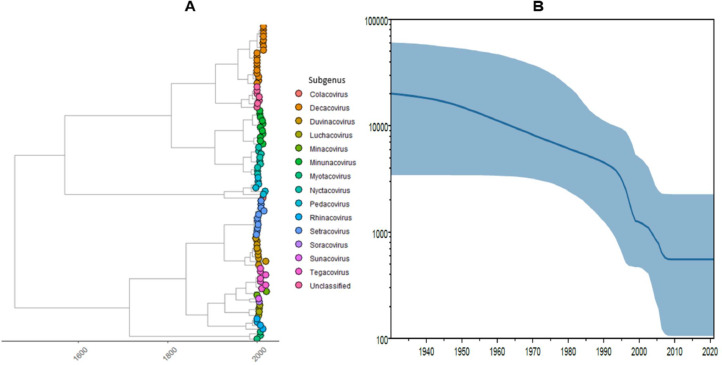
**(A)** Time scaled Maximum clade credibility tree (MCC) of ORF1ab region of AlphaCoV. Based on the legend, branch colours denote AlphaCoV subgenera, while branch lengths correspond to time in years, **(B).** Bayesian Skyride plot of All AlphaCoV analysed in this study showing estimates of the effective virus population size over time. The upper and lower blue lines represent the population's 95% high posterior density intervals (95% HPD), while the solid blue line depicts the median population size.

To investigate the origin and timing of the Nigerian AlphaCoV lineage's emergence and establishment, we performed phylogeographic reconstruction of our three full genomes along with 91 AlphaCoV reference of all the known AlphaCoV subgenera. Our phylogeographic reconstruction was limited to the ORF1ab region since it is the genomic region used by ICTV for species demarcation. We estimate that the unclassified AlphaCoV strains in this study seemed to evolve out of its MRCA about 70 years ago with a TMRCA of 1950 (95% HPD, 1920–1980) and evolved at a rate of 9.96×10^−5^ substitution per site per year (95% HPD 1.63×10^−5^ – 3.31×10^−4^) (Fig. 2a). The Nigerian sequences clustered monophyletically consistently (similar to the ML tree), which further supported the suggestion that the lineage may have been established by a single introduction. We found that with the Bayesian model, the Nigerian sequences formed a sister lineage to a group of sequences isolated in bats in the *Molossidae* family as previously shown in the ML tree. We also observed that members in the *Decacovirus* subgenus may have shared a common ancestor with the unclassified AlphaCoV in the past, and had the earliest ancestral diversification from the parental lineage with a TMRCA of 1948 (95% HPD 1900 – 1990).

To reconstruct changes in population sizes of AlphaCoV over time and acquire useful insights into various evolutionary and population-genetic processes, we performed a Bayesian Skyride analysis involving all AlphaCoV subgenera. We show that the AlphaCoV effective viral population size appear to be on a decline in the past until 2010 where it appears to have reached equilibrium prevalence (Fig. 2b).

### 3.3 Recombination analysis result

**Fig. 3:**
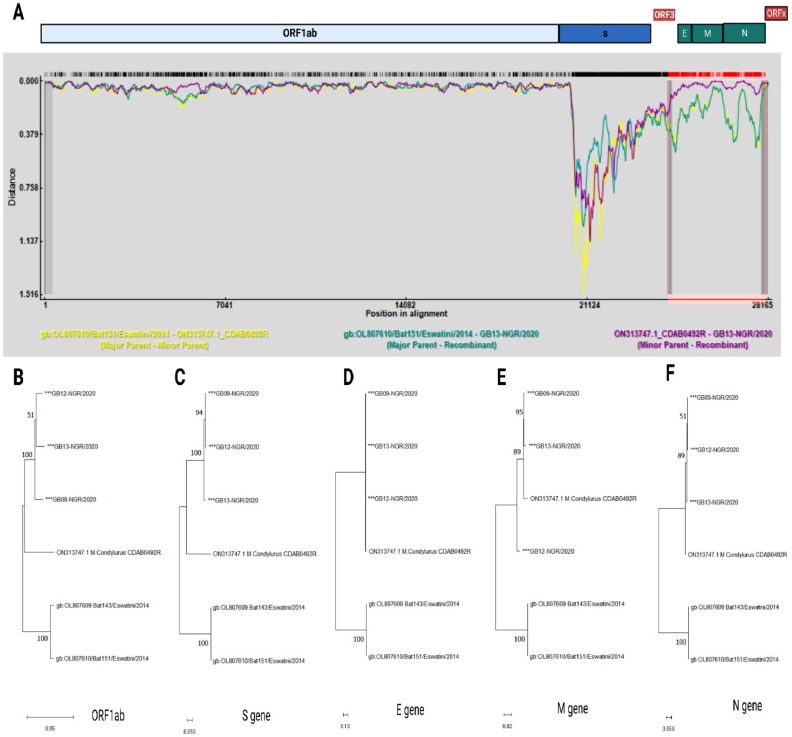
Genomic analyses of putative recombinant alphacoronavirus with evidence of recombination at breakpoints 24,032–27,736. (**A**) Distance Plot analyses of GB013-NGR_2020 from this study predicted to be a recombinant of *Chaerephon pumilus* bats alphacoronavirus (OL807610-Major Parent highlighted in green) and unverified Alphacoronavirus (ON313747.1-Minor Parent highlighted in purple) while the figures below shows the Maximum likelihood tree of AlphaCoV detected in bats in *Molossidae* family based on **B**. complete ORF1ab protein, **C.** Spike protein, **D.** Envelope protein, **E.** Membrane protein and **F**. Nucleocapsid protein, with 1000 bootstrap replications. The Nigerian strains reported in this study are asterisked.

One unique recombination event involving members of the unclassified AlphaCoV detected in *Molossidae* bats was seen in the analysis of complete genome sequences by at least six of the nine statistical methods offered by RDP4. Isolate GB013-NGR_2020 from this study was predicted to be a recombinant of *Chaerephon pumilus* bats alphacoronavirus (Bat151/Eswatini/2014 [OL807610]) and unverified Alphacoronavirus (CDAB0492R [ON313747.1]) (Fig. 3A). Maximum likelihood trees of members of the unclassified AlphaCoV detected in *Molossidae* bats using the predicted protein sequences of ORF1ab, S, E, M, and N showed topology incongruence at the M protein, which lies within the 24,032–27,736 breakpoint region (Fig. 3B-F).

### 3.4 Spike (S) protein sequence analysis results

According to the ProtParam analysis, the Nigerian strain's S protein contains 1357 (GB09-NGR-2020 and GB012-NGR-2020) and 1355 (GB013-NGR-2020) amino acids with molecular weights ranging from 148,667.44–149,079.93 and isoelectric points ranging from 6.27–6.50, respectively. Each of their proteins had 96 negatively charged residues and 89–91 positively charged residues. A complete list of ProtParam-generated results for all unclassified AlphaCoVs, including Bat151/Eswatini/2014 and CDAB0492R/DRC is shown in Supplementary Table 2. The results using Predicting Antigenic Peptide software showed that GB09-NGR-2020 S protein has 61 epitopes, GB013-NGR-2020 S protein has 60 epitopes, Bat151/Eswatini/2014 S protein has 54 epitopes, and CDAB0492/DRC/2018 S protein has 54 epitopes (supplementary fig. 3A-D)

**Fig. 4:**
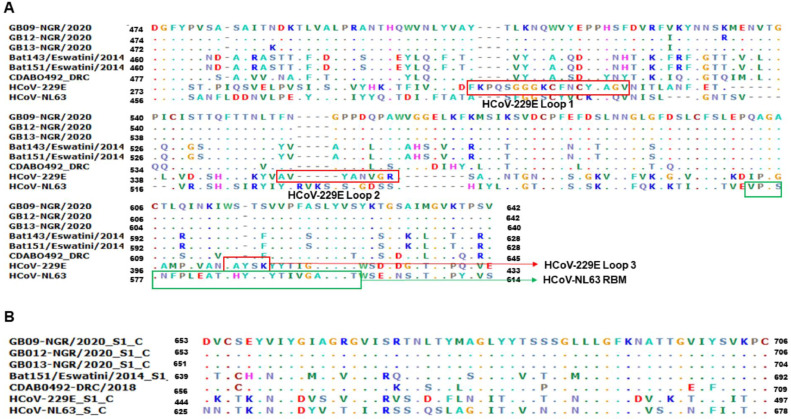
Sequence alignment showing amino acid similarities and variations in **A.** S-RBD and **B.** S1 C-terminal domain of the unclassified AlphaCoV in comparison with two human Alphacoronavirus HCoV-NL63 and HCoV-229E. The highlighted red section in the alignment denotes the receptor binding motifs of HCoV-229E that contain most of the contacting residues that bind to hAPN while the highlighted section in green denotes HCoV-NL63 binding motifs (amino acid residues 573–599) that contain contacting residues that bind to ACE2. The dot in the alignment denotes amino acid similarities.

To gain insight into the amino acid similarities and variations in the S-RBD, S1 C-terminal domain and HR (1 and 2), of the unclassified AlphaCoV in comparison with HCoV-NL63 and HCoV-229E which might indicate a possibility of binding to human receptors, we observed that the unclassified AlphaCoVs have a distinctive RBD different from the contacting amino acid residues that bind to both ACE2 and hAPN (fig. 4a). Precisely, in the receptor binding motifs of HCoV-229E that contain most of the contacting residues that bind to hAPN (Loop 1 [amino acid residues 308–325]; Loop 2 [amino acid residues 352–359]; Loop 3 [amino acid residues 404–408]), only P322 (Loop 1) and W404 (Loop3) were the only observed amino acid residues that showed similarity to the Nigerian strain. For HCoV-NL63 binding motifs (amino acid residues 573–599) that contain contacting residues that bind to ACE2, only 29.6% (8/27) of the amino acid residues were similar to the unclassified AlphaCoV. However, for the C-terminal region of the spike protein, the aligned coronaviruses shared significant residues due to the conserved nature of the region (Fig. 4B).

Sequence alignment of Spike heptad repeat 1 and 2 (HR1 and HR2) from unclassified bat AlphaCoV and human AlphaCoVs (HCoV-NL63 and HCoV-229E) revealed various substitutions and conserved regions in both HR1 and HR2. Specifically, we observed four amino acid substitutions in the HR1 region (K987S, D1001S, D1036M and T1040A) and one substitution (S1230L) in the HR2 region that were unique to the Nigerian strains detected in this study (supplementary fig. 4).

## 4 Discussion

In this study, we add three near-complete genome sequences of alphacoronaviruses from *Mops condylurus* (Angolan free-tailed bat) species (a member of the *Molossidae* family) to the public NCBI repositories which is the first report from Nigeria. Members in the *Molossidae* family are one of Africa's frequently described bat species which forage in urban and agricultural environments (Happold, 2013; Noer et al., 2012), with some seen roosting around human dwellings (George et al., 2022; Goldstein et al., 2018).

This study also demonstrated the utility of metagenomic deep sequencing in recovering larger portions of a novel CoV genome, and also enabling extensive characterization and evaluation of the CoV genome. However, the absence of AlphaCoV reads in some samples that were previously reported to be PCR positive for AlphaCoV may be due to CoV RNA degradation, or inefficiencies in library preparation reactions. A major disadvantage of the technique is that it requires a large quantity of genomic starting material (e.g., a high viral load). Furthermore, due to the ability to amplify any DNA or RNA genome at random (including commensal microorganism and host genome DNA), minority genetic material may be excluded or even lost (low sensitivity) (Ibañez-Lligoña et al., 2023). Based on our findings, we suggest that the most efficient CoV identification and surveillance programs will combine deep metagenomic sequencing, amplicon sequencing and probe capture. Although the feasibility of this approach in a resource-limited environment might be a challenge.

The analysis of the pairwise identity of five ORFs of AlphaCoV in this study to those of previously described AlphaCoVs subgenera showed similarity only to unclassified AlphaCoV detected in Members from the *Molossidae* bat family (Kuchinski et al., 2022; Shapiro et al., 2021; Tao et al., 2012). Using the coronavirus ICTV demarcation criteria, we were able to categorize species members of unclassified AlphaCoVs from the *Molossidae* bat family as potentially unique subgenera. Interestingly, the AlphaCoV strains detected in *Mops condylurus* bats in this study formed a genetic cluster with AlphaCoVs previously detected exclusively in bats from members of the *Molossidae* family in the Democratic Republic of Congo, Eswatini, and China. This suggests significant cryptic transmission of AlphaCoVs within members of the *Molossidae* family, with robust genome-wide surveillance likely to reveal ongoing transmission of divergent lineages. Co-evolution and host switching have been identified as crucial evolutionary mechanisms for CoVs in Africa and Asia (Latinne et al., 2020; Maganga et al., 2020; Tong et al., 2009). However, further studies are necessary to explain the predilection of these AlphaCoVs for species in the *Molossidae* family to comprehend the process of host tropism, especially the host receptor use requirements of the AlphaCoVs, the likelihood of interspecies transmissibility to other bat species, and the role that bat ecology plays in this phenomenon. There is also a need to investigate the role of coevolutionary forces in making the unclassified AlphaCoV more adaptable to its host.

The estimated mean substitution rate showed that the unclassified AlphaCoV has a lower mutation substitution rate than SARS-CoV-2, HCoV-OC43, and MERS-CoV (Bukin et al., 2021; Vijgen et al., 2005; Wang et al., 2022; Zhang et al., 2016). The reason for this could be that the unclassified AlphaCoV has a robust proofreading mechanism that can correct some errors that may arise during the replication process, resulting in a decrease in the AlphaCoV's mutation rate (Minskaia et al., 2006). From our analysis, the unclassified AlphaCoV strains in this study seemed to have evolve out of their most recent common ancestor (MRCA) more than 70 years ago. They were not detected till around 2009 in Kenya from *Chaerephon spp.* collected in 2006 and more recently in 2021/2022 from *Mops condylurus* bats because of infrequent surveillance (George et al., 2022; Kuchinski et al., 2022; Shapiro et al., 2021; Tong et al., 2009).

The demographic history of bat AlphaCoV sequences revealed a declining population demography, possibly due to genetic drift. In theory, genetic drift has a significant impact on the frequency and fate of mutations in populations with a small effective population size (Moya et al., 2004; Rouzine et al., 2001). Other factors, such as genetic bottleneck (particularly during intra- or inter-species transmission) and variation in replication potential among variants, could lead to a decrease in effective population size (Moya et al., 2004).

Recombination events involving AlphaCoVs strains detected in members of the *Molossidae* family (*Mops condylurus* and *Chaerephon pumilus*) were detected, suggesting that the virus may be using bat species in this family as mixing vessels to generate CoVs that can adapt to a new host. Furthermore, GB013-NGR_2020 might acquire/provide its M protein from/to other CoVs through such recombination to present novel virulence features. Although recombination is common in similar CoV species, it occurs rarely among different CoV species to generate novel viruses. The coronavirus M protein is essential for virion morphogenesis with an active role in virus assembly, transforming cellular membranes into hotspots where virus and host factors interact to create new virus particles (Neuman et al., 2011; Siu et al., 2008). Missense mutations in the M gene, on the other hand, have been reported to be relatively uncommon, most likely due to purifying selection (Cagliani et al., 2020). However, as a result of the constant exposure of M gene to evolutionary constraints, CoVs carrying M gene mutations and suggested to be biologically fit with rapid evolution potential have been reported (Shen et al., 2021). Other investigations of coronavirus recombination have similarly discovered various recombination breakpoints (de Klerk et al., 2022; Pollett et al., 2021), indicating a high frequency of recent homologous recombination between coronavirus strains.

The coronavirus S proteins have been known to be highly variable within the CoV genome and coronavirus S proteins are more closely related to members of the same group or subgroup than to members of a different group or subgroup. A startling distinction was found between the pairwise identity of the S gene sequences in this study and the S gene of other alphacoronaviruses (≤77% amino acid identities to the S proteins of CoV in other subgenera), indicating that the Nigerian strains may have a genetically unique spike protein that is only distantly related to other AlphaCoVs. Strong selective pressure may have caused the Nigerian strains to rapidly evolve their S protein, or they may have acquired this unique S protein through recombination with an unidentified coronavirus. The S protein of CoVs is frequently the target of selection pressure because it is involved in receptor binding and has epitopes for antibodies that can neutralize it (Berry et al., 2010; Du et al., 2009; Mittal et al., 2022).

The unclassified AlphaCoVs had very low amino acid identities to the corresponding regions that bind to hAPN and ACE2, based on an analysis of contacting amino acid residues of HCoV-229E and HCoV-NL63-S proteins. This implies that the unclassified AlphaCoVs have binding sites that are distinct from the motifs used for ACE2 and hAPN binding. Changes in the RBM region of the HCoV-NL63 S protein (which is similar to the RBM region of SARS-CoV) have been shown to interfere with ACE2 binding (Li et al., 2007). We observed slight variations in both the HR1 and HR2 regions involving non-polar to polar amino acid substitution. Mutation in the HR1 domain of the spike fusion protein has been associated with escape from HR-HR2-derived entry inhibition (Bosch et al., 2008). To fully understand the spike protein's function in evolution and interspecies transmission, structural studies are required.

Using Predict Antigenic Peptides, we identified several antigenic epitopes for the unclassified AlphaCoVs S proteins (supplementary fig. 2A-D). This prediction will guide future research on the use of these epitopes in antiviral inhibitor discovery.

## 5 Conclusion

In conclusion, this is the first report of near-complete genomes of bat alphacoronavirus in Nigeria that originated from *Mops condylurus* bats previously captured from the ceiling of a residential building. This will guide the development of tools to understand better the epidemiology and surveillance of bat CoVs in Nigeria and Africa, where only a few short genome segments of bat CoVs have been reported. The current study also provides information on the interspecies transmission of CoVs between bat species members in the family, *Molossidae*, and it exemplifies the value of genome sequencing and analysis in comprehending coronavirus evolution. Given the wide range of bat species found in Africa, ongoing monitoring of non-human CoV hosts is necessary for the early detection of possible zoonotic outbreaks, particularly in Africa's under-sampled regions.

## CRediT authorship contribution statement

Conceptualization, U.G, O.G, O.F, AH, I.K. and C.H.; methodology, U.G., J.U, O.O and P.E; sample collection, U.G, O.G, and J.K; software and bioinformatics analysis, U.G. and B.M; resources, U.G., I.K and C.H; writing—original draft preparation, U.G.; writing—review and editing, J.K, O.G, B.M, J.O, O.O, A.H, O.F, I.K. and C.H.; visualization, U.G and B.M.; supervision, I.K. and C.H.; funding acquisition, U.G and C.H. All authors have read and agreed to the published version of the manuscript.

## Funding

This research was funded partially by the 2020 ISID grant to UG. This work was made possible by support from Flu Lab and a cohort of generous donors through TED's Audacious Project, including the ELMA Foundation, MacKenzie Scott, the Skoll Foundation, and Open Philanthropy. This work was supported by grants from the National Institute of Allergy and Infectious Diseases (https:// www. NIAID. NIH. gov), NIH-H3Africa (https:// h3afr ica. org) (U01HG007480, U54HG007480 and U01AI151812), the World Bank grants (project ACE-019 and ACE-IMPACT), the Rockefeller Foundation (Grant #2021 HTH), the Africa CDC through the African Society of Laboratory Medicine [ASLM] (Grant #INV018978), the Wellcome Trust (Project 216,619/Z/19/Z), and the Science for Africa Foundation.

## Data availability statement

Genome sequences of bat coronaviruses reported in this study have been deposited in GenBank under accession numbers OQ792153- OQ792170.

## Declaration of Competing Interest

The authors declare no conflict of interest.

## Data Availability

Data will be made available on request.

## Appendix Supplementary materials

Image, application 1
