# Supplementary material for: Genomic characterization of Alphacoronavirus from Mops condylurus bats in Nigeria
Source: Virus Res. 2023 Jul 24;334:199174. doi: 10.1016/j.virusres.2023.199174 (PMC10392604; doi:10.1016/j.virusres.2023.199174)
Supplement: Supplementary file 1 [file mmc1.docx]

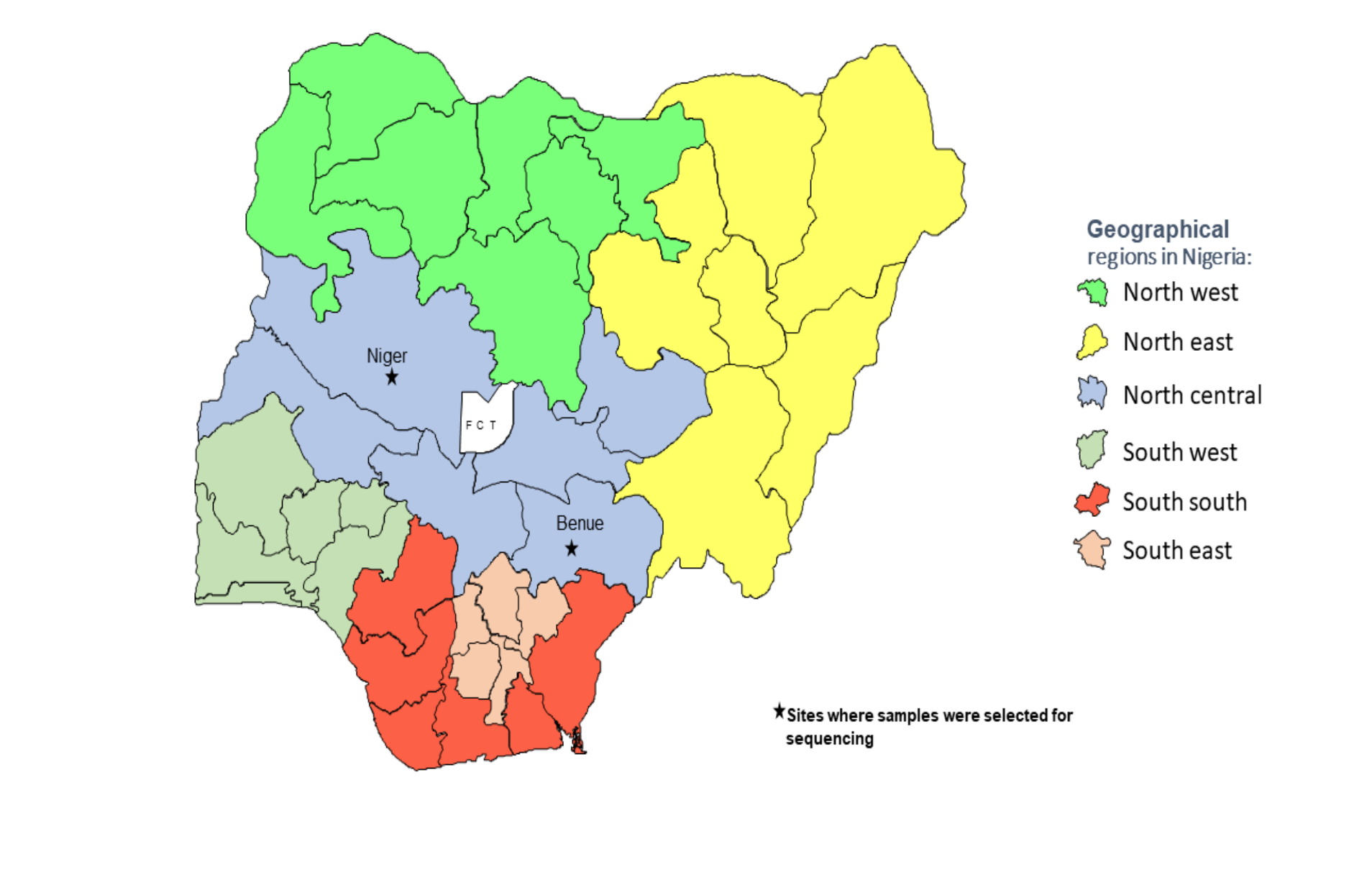
**Supplementary Figure 1.** Map of Nigeria showing the various regions AlphaCoVs were detected and subsequently sequenced in this study.


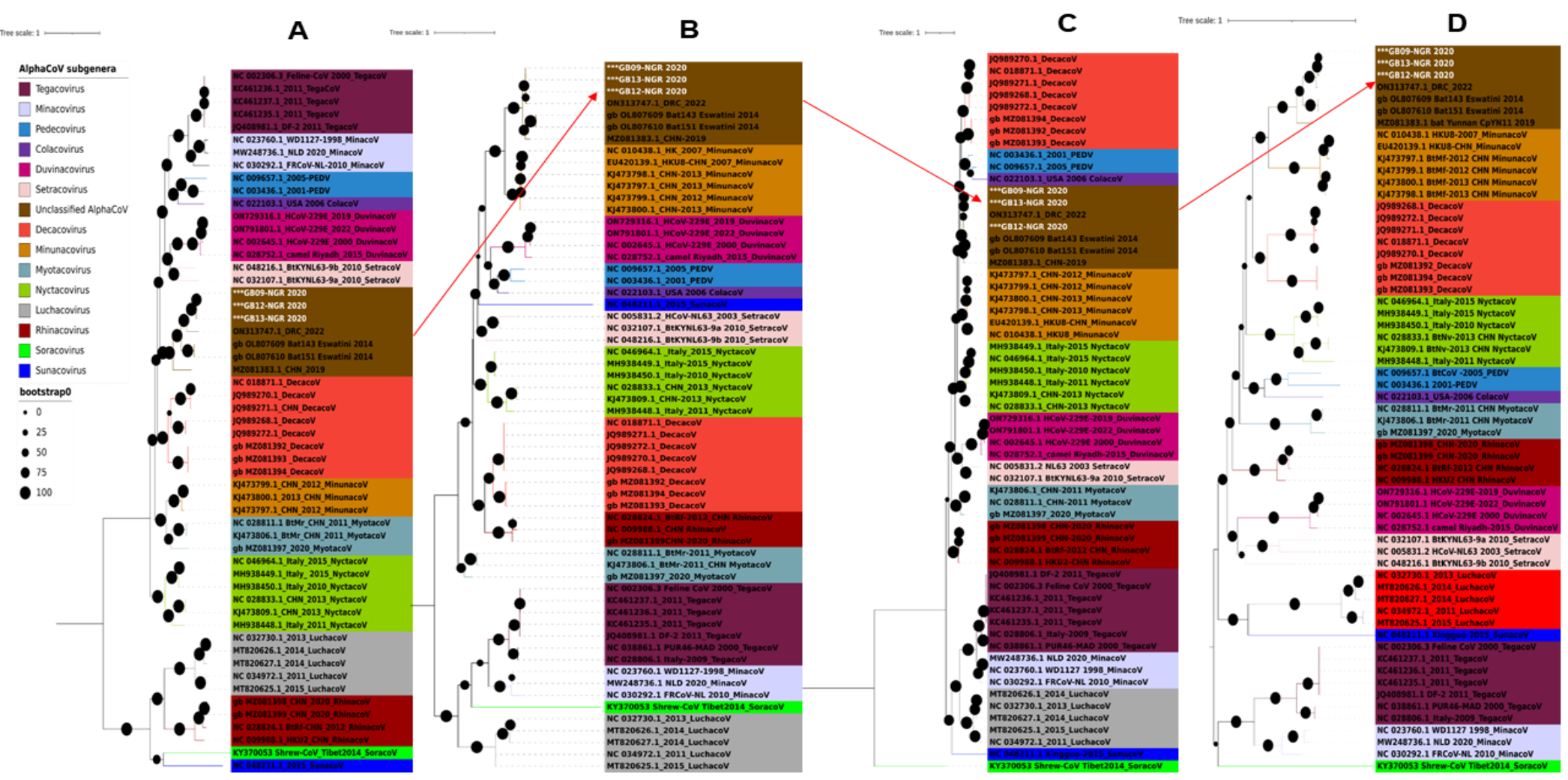


**Supplementary Figure 2.** Maximum likelihood tree of AlphaCoV detected in bats in *Molossidae* family based on A. Spike protein, **B.** Envelope protein, **C.** Membrane protein and **D**. Nucleocapsid protein, with 1000 bootstrap replications. AlphaCoV subgenera are colour-coded as shown in the legend and the Nigerian strains reported in this study are asterisked and highlighted in white. The Interactive Tree of Life (iTOL) v5 with midpoint rooting was used to visualise the tree. The Best-fit model according to BIC was GTR+F+I+G4

**Supplementary Table 1:** Accession number, host, subgenera and country of sequences analyzed in this study

| Isolate/Accession number | Host | AlphaCoV subgenera | Country |
| --- | --- | --- | --- |
| GB09-NGR/2020 | Bat-Mops condylurus | Unclassified | Nigeria |
| GB012-NGR/2020 | Bat-Mops condylurus | Unclassified | Nigeria |
| GB013-NGR/2020 | Bat-Mops condylurus | Unclassified | Nigeria |
| ON313747.1 | Bat-Mops condylurus | Unclassified | Democratic Republic of Congo |
| MZ081383.1 | Bat-Chaerephon plicatus | Unclassified | China |
| OL807609 | Bat-Chaerephon pumilus | Unclassified | Eswatini |
| OL807610 | Bat-Chaerephon pumilus | Unclassified | Eswatini |
| MK720945.1 | Bat | Decacovirus | Hong Kong |
| MK720946.1 | Bat | Decacovirus | Hong Kong |
| MZ081385 | Bat | Decacovirus | China |
| MZ081386 | Bat-Myotis_muricola | Decacovirus | China |
| MZ081384 | Bat-Myotis_muricola | Decacovirus | China |
| MZ081387 | Bat-Myotis_muricola | Decacovirus | China |
| MZ081388 | Bat-Hipposideros_larvatus | Decacovirus | China |
| MZ081392 | Bat-Murina_cyclotis | Decacovirus | China |
| MZ081393 | Bat-Rhinolophus_malayanus | Decacovirus | China |
| MZ081394 | Bat-Rhinolophus_malayanus | Decacovirus | China |
| NC_018871.1 | Bat | Decacovirus | China |
| JQ989270.1 | Bat | Decacovirus | China |
| JQ989271.1 | Bat | Decacovirus | China |
| JQ989266.1 | Bat | Decacovirus | Hong_Kong |
| JQ989267.1 | Bat | Decacovirus | Hong_Kong |
| JQ989268.1 | Bat | Decacovirus | Hong_Kong |
| JQ989269.1 | Bat | Decacovirus | Hong_Kong |
| JQ989272.1 | Bat | Decacovirus | Hong_Kong |
| NC_010437.1 | Bat | Minunacovirus | Hong_Kong |
| EU420138.1 | Miniopterus_pusillus | Minunacovirus | Hong_Kong |
| KJ473795.1 | Miniopterus_fuliginosus | Minunacovirus | China |
| KJ473796.1 | Miniopterus_fuliginosus | Minunacovirus | China |
| EU420137.1 | Miniopterus_pusillus | Minunacovirus | Hong_Kong |
| NC_010438.1 | Miniopterus_bat | Minunacovirus | Hong_Kong |
| EU420139.1 | Miniopterus_pusillus | Minunacovirus | Hong_Kong |
| KJ473797.1 | Miniopterus_fuliginosus | Minunacovirus | China |
| KJ473798.1 | Miniopterus_fuliginosus | Minunacovirus | China |
| KJ473799.1 | Miniopterus_fuliginosus | Minunacovirus | China |
| KJ473800.1 | Miniopterus_fuliginosus | Minunacovirus | China |
| NC_046964.1 | Bat-Pipistrellus_kuhlii | Nyctacovirus | Italy |
| MH938449.1 | Bat-Pipistrellus_kuhlii | Nyctacovirus | Italy |
| MH938450.1 | Bat-Pipistrellus_kuhlii | Nyctacovirus | Italy |
| NC_028833.1 | Bat-Nyctalus_velutinus | Nyctacovirus | China |
| KJ473809.1 | Bat-Nyctalus_velutinus | Nyctacovirus | China |
| MH938448.1 | Bat-Pipistrellus_kuhlii | Nyctacovirus | Italy |
| MK472069.1 | Bat-microbat | Nyctacovirus | AUSTRALIA |
| MK472068.1 | Bat-microbat | Nyctacovirus | AUSTRALIA |
| MK472071.1 | Bat-microbat | Nyctacovirus | AUSTRALIA |
| NC_022103.1 | Myotis_lucifugus | Colacovirus | USA |
| NC_009657.1 | Scotophilus_bat | Pedacovirus | China |
| NC_003436.1 | Scotophilus_bat | Pedacovirus | China |
| OQ915150 | Sus_scrofa | Pedacovirus | China |
| MZ313556 | Sus_scrofa | Pedacovirus | Poland |
| MZ325484 | Sus_scrofa | Pedacovirus | Poland |
| MZ313557 | Sus_scrofa | Pedacovirus | Poland |
| NC_028752.1 | camel | Duvinacovirus | Saudi_Arabia |
| NC_002645.1 | Human | Duvinacovirus | Saudi_Arabia |
| ON554133 | Human | Duvinacovirus | China |
| ON554134 | Human | Duvinacovirus | China |
| ON729316.1 | Human | Duvinacovirus | USA |
| ON791801.1 | Human | Duvinacovirus | USA |
| MT438698 | Human | Duvinacovirus | USA |
| MT438696 | Human | Duvinacovirus | USA |
| MT438697 | Human | Duvinacovirus | USA |
| NC_005831.2 | Human | Setracovirus | Netherlands |
| LC756668 | Human | Setracovirus | Japan |
| LC687397 | Human | Setracovirus | Japan |
| MW202337 | Human | Setracovirus | USA |
| LC687402 | Human | Setracovirus | Japan |
| MN306018 | Human | Setracovirus | USA |
| ON553985 | Human | Setracovirus | China |
| ON553965 | Human | Setracovirus | China |
| MG428701 | Human | Setracovirus | Kenya |
| MG428703 | Human | Setracovirus | Kenya |
| MG428702 | Human | Setracovirus | Kenya |
| NC_028811.1 | Bat-Myotis_ricketti | Myotacovirus | China |
| KJ473806.1 | Bat-Myotis_ricketti | Myotacovirus | China |
| MZ081397 | Bat-Myotis_laniger | Myotacovirus | China |
| NC_028824.1 | Bat-Rhinolophus_ferrumequinum | Rhinacovirus | China |
| MZ081398 | Myotis_laniger | Rhinacovirus | China |
| MZ081399 | Rhinolophus_stheno | Rhinacovirus | China |
| NC_009988.1 | Rhinolophus_stheno | Rhinacovirus | China |
| NC_023760.1 | Mustela_vison | Minacovirus | USA |
| MW248736.1 | Neovison_vison | Minacovirus | Netherlands |
| NC_038861.1 | Pig | Tegacovirus | USA |
| NC_028806.1 | Pig | Tegacovirus | Italy |
| NC_002306.3 | Pig | Tegacovirus | USA |
| KC461235.1 | Pig | Tegacovirus | USA |
| KC461237.1 | Pig | Tegacovirus | USA |
| JQ408981.1 | Pig | Tegacovirus | USA |
| KC461236.1 | Pig | Tegacovirus | USA |
| MT820625.1 | Chevriers_field_mouse | Luchacovirus | China |
| NC_032730.1 | Rattus_norvegicus | Luchacovirus | China |
| MT820626.1 | Rattus_norvegicus | Luchacovirus | China |
| MT820627.1 | Eothenomys_miletus | Luchacovirus | China |
| NC_048211.1 | Shrew-Suncus_murinus | Sunacovirus | China |
| KY370053 | Shrew-Sorex_araneus | Soracovirus | China |

**Supplementary Table 2: Physical and Chemical properties of Unclassified AlphaCoV spike proteins from *Molossidae* members.**

| **Characteristic** | **GB09-NGR-2020** | **GB012-NGR-2020** | **GB013-NGR-2020** | **Bat151/Eswatini/2014** | **CDAB0492R_DRC_2018** |
| --- | --- | --- | --- | --- | --- |
| Number of amino acids | 1357 | 1357 | 1355 | 1376 | 1376 |
| Formula | C_6670_H_10313_N_1743_O_1996_S_67_ | C_6672_H_10322_N_1746_O_1994_S_68_ | C_6645_H_10298_N_1754_O_1985_S_67_ | C_6749_H_10532_N_1778_O_1999_S_66_ | C_6771_H_10468_N_1780_O_2021_S_60_ |
| Molecular weight | 149004.76 | 149079.93 | 148667.44 | 150680.54 | 151067.91 |
| Theoretical isoelectric point (PI) | 6.27 | 6.30 | 6.50 | 6.73 | 6.02 |
| Number of negatively charged residues | 96 | 96 | 96 | 96 | 102 |
| Number of positively charged residues | 89 | 89 | 91 | 93 | 90 |
| Instability index (II) | 31.93 (stable) | 31.64 (stable) | 31.61 (stable) | 32.82 (stable) | 32.52 (stable) |
| Aliphatic index | 89.40 | 89.84 | 90.04 | 95.47 | 93.30 |
| Grand average of hydropathicity (GRAVY) | 0.101 | 0.106 | 0.096 | 0.169 | 0.098 |


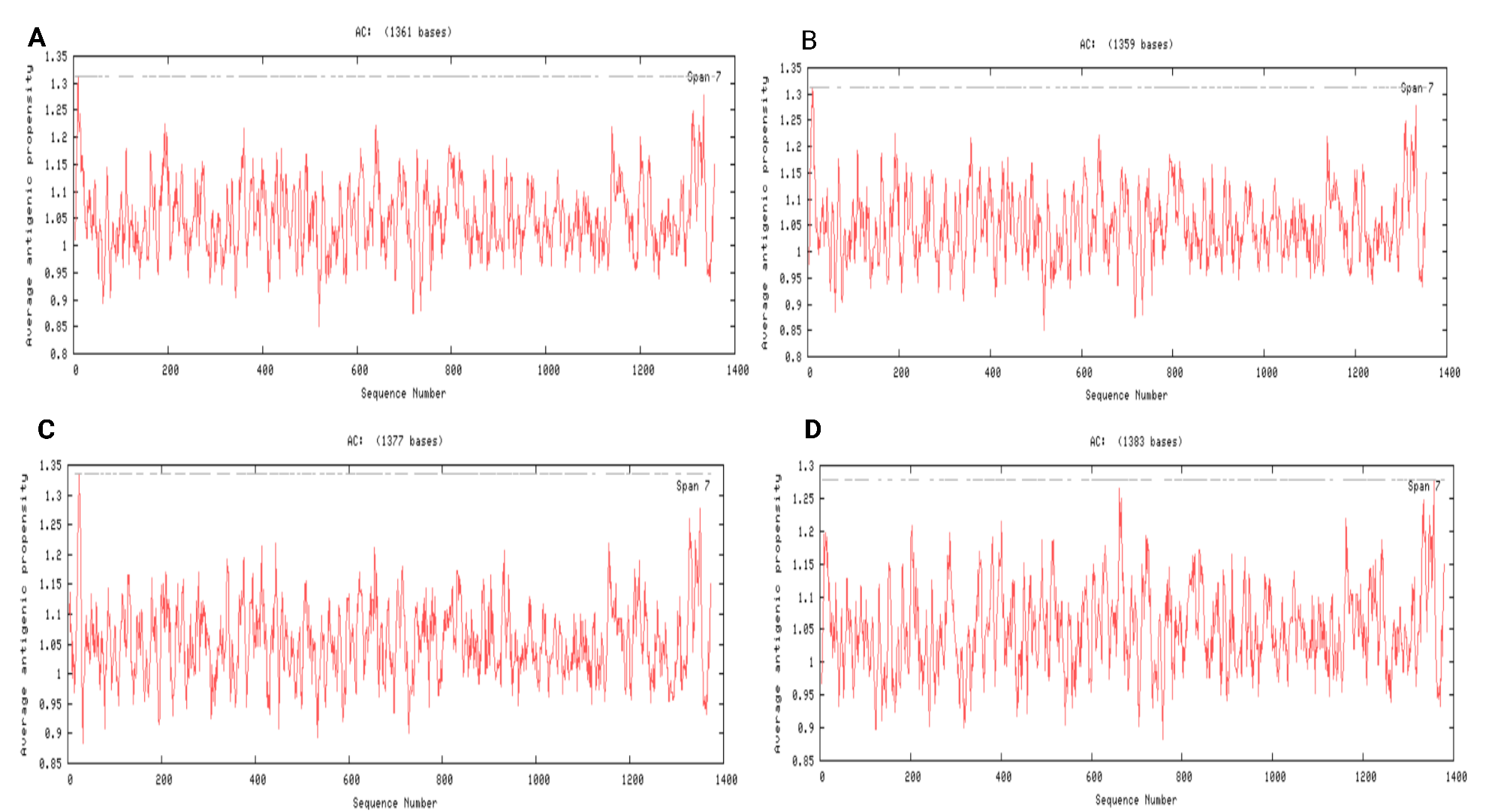


**Supplementary figure 3.** Analysis of antigenic epitopes. A. GB09-NGR-2020 S protein showing 61 epitopes, B. GB013-NGR-2020 S protein showing 60 epitopes, C. Bat151/Eswatini/2014 S protein showing 54 epitopes, and D. CDAB0492/DRC/2018 S protein showing 54 epitopes respectively.


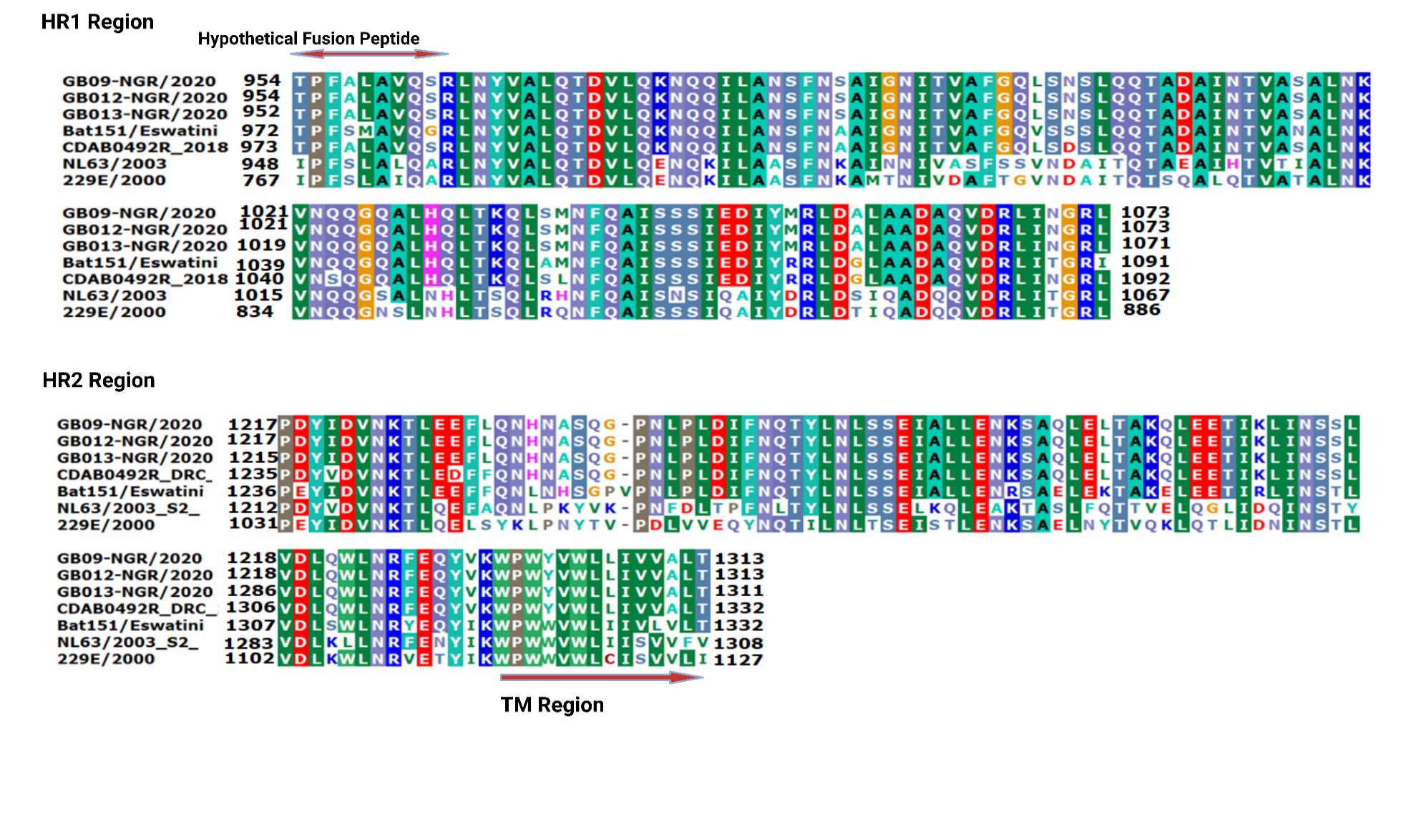


**Supplementary figure 4**: Sequence alignment of the Heptad Repeat region 1 and 2 of GB09-NGR-2020, GB012-NGR-2020, GB013-NGR-2020, Bat151/Eswatini/2014, CDAB0492R/DRC and two human Alphacoronavirus (HCoV-NL63 and HCoV-229E).
